# Primary omental torsion in a 9 year old girl: a case report

**Published:** 2014-06-25

**Authors:** D Anyfantakis, M Kastanakis, V Karona, EK Symvoulakis, G Fragiadakis, N Katsougris, E Bobolakis

**Affiliations:** * Primary Health Care Centre of Kissamos, Chania, Crete, Greece; ** First Department of Surgery, Saint George General Hospital of Chania, Crete, Greece; *** Private Family Practice Unit in Heraklion, Crete, Greece

**Keywords:** primary omental torsion, acute abdomen, diagnosis, management

## Abstract

Abstract

Primary torsion of the omentus is an extremely unusual cause of acute abdomen in the pediatric population. This condition occurs from twist of the pedicle of the omental apron around its longer axis, leading to edema, ischaemia and necrosis. Here we present a rare case of a 9 year old girl referred by her general practitioner due to severe right lower quadrant abdominal pain with a presumed diagnosis of acute appendiceal inflammation. Surgical operation disclosed primary omental torsion. The infarcted segment was resected and the girl’s clinical recovery was uneventful without any complication. The condition may mimic a variety of other causes of acute abdominal symptoms. In this case report, a presumed diagnosis of acute appendicitis urgently induced the decision of a surgical approach. Physicians involved in the acute pediatric care have to include this rare condition in the differential diagnosis of acute onset right-sided abdominal pain.

## Introduction

Primary omental torsion represents an extremely rare etiology of acute abdomen [**1**], especially among children [**2**]. It is remarkable that Kimber et al, in a 20 years retrospective study among a paediatric population reported that the ratio of primary omental torsion to appendicitis was found less than 4/1000 respectively [**1,3**]. In a similar 10 year study in Greece this ratio was found 1/587 with overweight males to be mainly affected [**2**]. 

 We report an unusual case of primary omental torsion in a 9 year old female. Information on clinical presentation, diagnosis and therapy is discussed.

## Case report

**Fig. 1:**
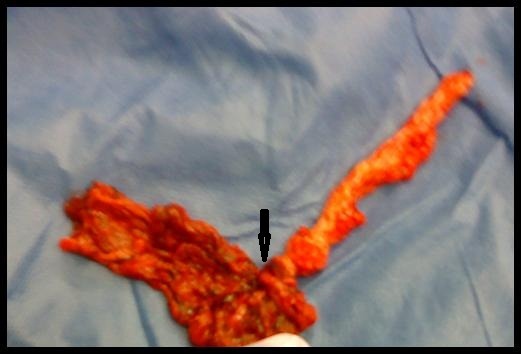
Intraoperative image of the rezected part of the omentus torsioned around a pivotal point (arrow)

**Fig. 2:**
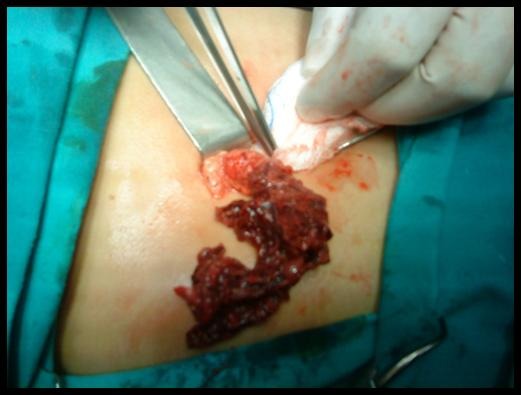
Intraoperative image of the omental part with evident signs of congestion and necrosis

A 9 year old girl was admitted to the Primary Health Care Centre of Kissamos complaining of acute onset abdominal pain for the last 6 hours located in the right lower quadrant. The girl was referred to the Emergency department of the Saint George General Hospital of Chania, Crete, Greece with the suspicion of acute appendiceal inflammation. Her vital signs on admission were within normal limits except of a slight elevation of her temperature (37.6 degrees Celsius). The girl’s weight was normal for her age. Physical examination revealed a marked right lower abdominal tenderness with guarding while obturator and psoas signs were negative. Intestinal sounds were normal. Initial laboratory work up included complete blood count, renal and liver function tests, urine analysis and chest X ray which were found normal. White blood cell count was 9500 cells/μl with 78% polymorphonuclears, 14% lymphocytes and 7% monocytes. The only pathological finding was a moderate elevation of the C-reactive protein levels [2.1 mg/dl (normal range: 0-0.5 mg/dl)]. Ultrasound (US) examination showed a small amount of free fluid in the pouch of Douglas. Visualization of the appendix was not feasible, while ovaries were found normal. Due to the increasing character of the abdominal pain, the girl was transferred to the operation room. Intraoperatively, during exploration of the peritoneal cavity was detected in the sero-sanguineous fluid. Appendix was found normal. Further exploration for Meckel’s diverticulum was negative. Torsion of the right omental part around its long axis (**Fig. 1** and **Fig. 2**) was discovered with evident signs of vascular congestion and necrosis (**Fig. 2**). Management consisted of resected of the twisted omentum. The girl recovered normally without any complications and she was discharged home 3 days later. 

## Discussion

Omental torsion was first described in 1899 by Eitel [**4**]. In regard to pathogenesis, torsion of the omentum around a pivotal point impairs its vascular perfusion resulting to severe congestion and oedema [1, 5]. Spontaneous derotation may occur at this stage [**6**]. However, if the torsion continues, edema progresses to acute hemorrhagic infarction and omental necrosis [**7**]. Extravasation of a sero-sanguineous liquid into the peritoneal cavity is often detected during surgery [**2,5**]. 

 The condition according to its aetiology is further classified as primary or secondary to certain predisposing risk factors [**1**]. Secondary omental torsion occurs more frequently than the primary form and is associated with intra-abdominal inflammatory situations, tumours or cysts [**8**]. Morris et al reported that the majority of secondary omental torsion cases are presented in patients suffering from inguinal hernia [**9**]. Regarding the primary or idiopathic form, it occurs in the absence of any intra-abdominal disorder [**5**]. 

A certain causative mechanism for primary omental torsion has not yet been established [**1**]. Intraoperatively, twist of the greater omentum around the right epiploic artery is often observed [**10**]. Increased mobility and length of the right side of the omentum may explain the increased prevalence of right omental infarction [**11**]. Factors that predispose a patient to torsion include congenital pathological variations of the omentum. Among these are reported bifid omentum which is an accessory omentum derived from a narrow route, abnormal embryological position of the right omental part with secondary fragile blood vessels [**12**] and irregular accumulation of omental fat [**1**]. Precipitating factors are those causing sudden omental displacement and include trauma, coughing, violent exercise, hyperperistalsis and compression between abdominal wall and the liver [**1**]. 

 Patients with omental torsion may present with a variety of non-specific symptoms and can mimic various aetiologies of acute abdomen [**1**]. Acute appendicitis, acute cholecystitis [**5**] and diverticulitis are conditions included in the differential diagnosis [**1**]. Right sided acute abdominal pain, fever, nausea and vomiting are the most frequently encountered symptoms at onset [**7,13**]. Physical examination reveals signs of peritoneal irritation with guarding and rebound abdominal tenderness [**14**]. A palpable abdominal mass has been also reported in rare cases [**13**]. Remarkably, patients with omental torsion share a more prolonged clinical course with less systematic manifestations compared to acute appendicitis [**14,15**].

 Although, US and Computed Tomography imaging are useful tools that assist the diagnosis by excluding acute appendicitis, cholecystitis and diverticulitis [**5,14**], the pathology is rarely diagnosed preoperatively, making exploratory laparotomy the most optimal diagnostic and therapeutic modality [**5**]. Surgical management of the primary omental torsion includes resection of the affected omental part with or without appendectomy [**16**]. Laparoscopic approach has been reported to be a safe and effective option [**14**]. Although conservative management has been described in some reports, surgery has been recommended as the treatment of choice in order to prevent severe complications related to conservative therapy such as sepsis and intra-abdominal abscess formation [**14**]. 

 In this case report, a presumed diagnosis of acute appendicitis urgently induced the decision of a surgical management. The intraoperative diagnosis was this of omental torsion. Describing, in detail, this episode of care which occurred in a 9 year old child, it is crucial to properly rule out causes of abdominal symptoms.

## Conclusions

We presented an unusual case of primary omental torsion in a non-obese 9 years old girl. Although accurate preoperative diagnosis is seldom feasible, physicians involved in the pediatric care have to consider this condition in cases of acute onset right-sided abdominal pain.

## References

[1] Scabini  S, Rimini  E (2011). Primary omental torsion: A case report. World J Gastrointest Surg.

[2] Mavridis  G, Livaditi  E (2007). Primary omental torsion in children: ten-year experience. Pediatr Surg Int.

[3] Kimber  CP, Westmore  P (1996). Primary omental torsion in children. J Paediatr Child Health.

[4] Eitel  CG (1899). Rare omental torsion. New York Med Rec.

[5] Andreuccetti  J, Ceribelli  C (2011). Primary omental torsion (POT): a review of literature and case report. World J Emerg Surg.

[6] Nihei  Z, Kojima  K (1991). Omental bleeding with spontaneously derotated torsion--a case report.. Jpn J Surg.

[7] Karayiannakis  AJ, Polychronidis  A (2002). Primary torsion of the greater omentum: report of a case. Surg Today.

[8] Leitner  MJ, Jordan  CG (1952). Torsion, infarction, and hemorrhage of the omentum as a cause of acute abdominal distress. Ann Surg.

[9] Morris  JH (1932). Torsion of the omentum. Arch Surg.

[10] Theriot  JA, Sayat  J (2003). Childhood obesity: a risk factor for omental torsion.. Pediatrics.

[11] Puylaert  JB (1992). Right-sided segmental infarction of the omentum: clinical, US, and CT findings. Radiology.

[12] Epstein  LI, Lempke  RE (1968). Primary idiopathic segmental infarction of the greater omentum: case report and collective review of the literature. Ann Surg.

[13] Efthimiou  M, Kouritas  VK (2009). Primary omental torsion: report of two cases. Surg Today.

[14] Tsironis  A, Zikos  N (2013). Acute abdomen due to primary omental torsion: case report.. J Emerg Med.

[15] Al-Jaberi  TM, Gharaibeh  KI (2000). Torsion of abdominal appendages presenting with acute abdominal pain. Ann Saudi Med.

[16] Young  TH, Lee  HS (2004). Primary torsion of the greater omentum. Int Surg.

